# A Rare Case of Spontaneous Tumor Lysis Syndrome in a Pulmonary Neuroendocrine Tumor

**DOI:** 10.7759/cureus.101859

**Published:** 2026-01-19

**Authors:** Jonathan L Graber, Marina Makram, Elie El Charabaty

**Affiliations:** 1 Internal Medicine, Touro College of Osteopathic Medicine, New York, USA; 2 Internal Medicine, Northwell Health, Staten Island University Hospital, Staten Island, USA; 3 Northwell Hofstra School of Medicine, Northwell Health, Staten Island University Hospital, Staten Island, USA

**Keywords:** acute kidney injury, bone biopsy, hepatic metastasis, pulmonary neuroendocrine tumor, spontaneous tumor lysis syndrome

## Abstract

Tumor lysis syndrome (TLS) is a serious medical condition in which the lysis of tumor cells results in a variety of abnormalities, including elevated uric acid, potassium, and phosphorus, decreased calcium, seizures, arrhythmias, and acute kidney injury (AKI). TLS most often occurs in hematologic cancers and after chemotherapy, whereas cases that arise without chemotherapy are termed spontaneous TLS (STLS). In our case, an 81-year-old female with a past medical history of hypertension and chronic obstructive pulmonary disease (COPD) initially presented to the emergency department for left lower back pain that was later found to have been caused by a bony metastatic lesion secondary to a neuroendocrine tumor of the lung. Her hospital stay was complicated by a worsening AKI that was refractory to fluids and the discontinuation of nephrotoxic medications. A uric acid, urine studies, lactate dehydrogenase, and metabolic panel ruled out other causes of her kidney injury and ruled in a diagnosis of STLS. The patient's condition worsened until she required hemodialysis, which she rejected in favor of comfort care. The patient died 14 days after admission. In conclusion, despite the majority of TLS cases originating from hematologic malignancies and in response to chemotherapy, STLS should be considered in refractory AKI for cancer patients, even when secondary to solid tumors like neuroendocrine tumors of the lung.

## Introduction

Tumor lysis syndrome (TLS) is a serious medical condition in which the lysis of tumor cells results in a variety of abnormalities, including elevated uric acid, potassium, and phosphorus, decreased calcium, seizures, arrhythmias, and acute kidney injury (AKI) [1]. TLS most often occurs in hematologic cancers and after chemotherapy, whereas cases that arise without chemotherapy are termed spontaneous TLS (STLS) [1,2]. Most cases of STLS have been reported in tumors with a high metastatic burden and thus increased cellular turnover [2]. Cases of STLS are reported to have higher mortality rates than regular TLS [2,3]. Furthermore, cases of TLS secondary to solid tumors have been reported to have greater mortality rates than TLS secondary to hematologic malignancies [4]. Therefore, it appears likely that STLS secondary to solid tumors is the most dangerous form of TLS.

The diagnostic criteria used for a formal diagnosis of laboratory TLS are known as the Cairo-Bishop criteria [5]. The criteria include at least two lab abnormalities in serum potassium, phosphorus, uric acid, and calcium within three days before or seven days after chemotherapy. Laboratory abnormalities are defined as a value that is outside the accepted normal range, or a 25% change from baseline. Clinical TLS requires fulfillment of laboratory TLS plus at least one of the following: arrhythmias, seizures, and AKI. There are no clear diagnostic criteria for STLS; therefore, we will rely on the Cairo-Bishop criteria and exclude the aspects relating to chemotherapeutic administration timing.

Treatment of TLS includes rapid fluid resuscitation in order to improve intravascular volume and urinary output, thereby increasing renal excretion of potassium, phosphorus, and uric acid [6]. Other interventions in the management of TLS are prescription of the hypouricemic agent rasburicase, renal replacement therapy, and correction of electrolyte imbalances [6]. Hyperkalemia should be treated quickly and aggressively, as its presence is the most hazardous acute complication that can cause sudden death from cardiac arrhythmias [6]. Treatment of hypocalcemia is reserved for patients with EKG changes or symptoms of neuromuscular irritability. In patients who are refractory to medical management of electrolyte abnormalities or with severe cardiac and neurologic manifestations, hemodialysis is recommended [6]. The best treatment for TLS is prevention; correctly identifying those at greatest risk and beginning monitoring are of the utmost importance [7]. For patients with hematologic malignancies starting chemotherapy, patients should be routinely monitored for TLS-relevant labs and adequately hydrated prior to chemotherapy [7]. Prophylactic rasburicase, if uric acid is already elevated, or allopurinol, if uric acid is normal, is often recommended for high-risk patients starting chemotherapy [7]. Owing to its relative rarity, no standardized monitoring or prophylactic protocol has been established for the primary prevention of STLS in patients with solid tumors.

## Case presentation

**Figure 1:**
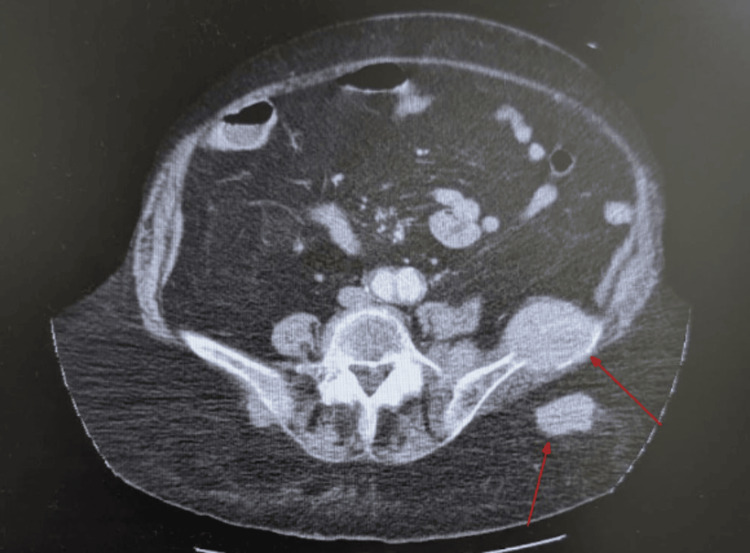
CT with IV contrast depicting bony and buttock metastatic lesions Soft tissue mass at the left iliac wing measuring 4.6 x 5.4 cm with erosive changes and a left buttock mass measuring 2.4 x 4.0 cm. Interventional radiology-guided biopsy was performed on the iliac lesion, and pathology confirmed the diagnosis of a high-grade neuroendocrine carcinoma.

**Figure 2:**
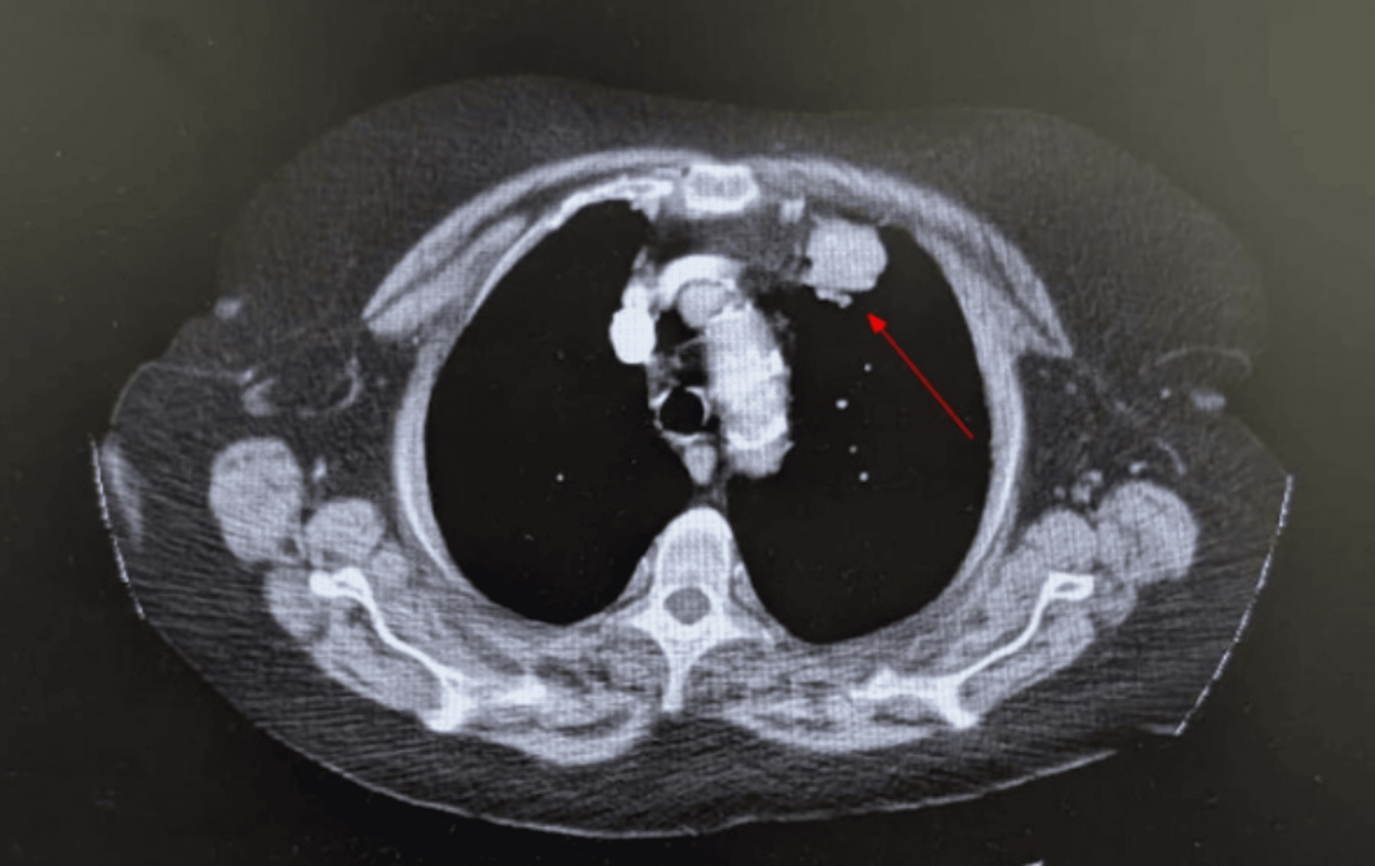
CT with IV contrast depicting lung mass Left apical lung mass measuring up to 2.9 cm. This was the presumed primary tumor based on biopsy results from the iliac lesion.

An 81-year-old white female with a past medical history of hypertension and chronic obstructive pulmonary disease initially presented to the emergency department for left lower back pain and a rash beneath her breasts. Her rash was determined to be candidiasis with superimposed cellulitis and was treated with oral Augmentin, fluconazole, and silver sulfadiazine cream. CT revealed metastatic lesions in her left iliac crest and buttock (Figure 1) in addition to prominent bilateral axillary lymph nodes. The primary cancer was at that time of unspecified origin. Additionally, there was evidence of smaller hepatic and thyroid metastatic lesions. The patient underwent a biopsy of the bony metastatic lesion in her left iliac crest. During her hospital stay, the patient developed worsening AKI as her creatinine rose by more than 0.3 above baseline. At that time, differential diagnoses included prerenal AKI, contrast-induced nephropathy (CIN), acute tubular necrosis (ATN), and TLS. However, a panel of labs returned reflective of TLS (Table 1) and, along with concurrent imaging tests, seemed to rule out other causes of AKI, or at least make them less likely. An ultrasound of the kidneys and bladder revealed a right-sided staghorn calculus without the presence of hydronephrosis in either kidney, making a postrenal AKI less likely. Additionally, the patient’s blood urea nitrogen (BUN)-to-creatinine ratio from day 3 onwards was consistently less than 20:1 (Table 1), which makes a prerenal AKI less likely. Therefore, it appeared the patient was suffering from an intrarenal AKI most likely caused by TLS, as indicated by her lab results, and less likely CIN or ATN. A urinalysis identified 13 casts in the urine but was unable to classify them. During her admission, the patient's creatinine continued to rise, and her biopsy results returned positive for a neuroendocrine tumor, likely of the lung, consistent with a lung mass seen on chest CT (Figure 2). The tumor was positive for CD56, synaptophysin, AE1/AE3, CAM 5.2 and negative for chromogranin, TTF-1/Napsin, CD45, S100. Ki-67 was very high. Altogether, the pathologist concluded the tumor is likely of neuroendocrine origin. Furthermore, the pathologist conjectured that the tumor likely originated in the lung or metastasized to the lung from another primary source. The absolute confirmation could not be made based on the sample they had. Therefore, the tumor was characterized as at least a tumor-node-metastasis (TNM) stage of IVB or extensive stage disease.

**Table 1:** Relevant lab values Based on the Cairo-Bishop Criteria [5] (minus the timing of chemotherapeutic administration), a diagnosis of both laboratory and clinical TLS would have been appropriate by day six. Treatment was deferred to day nine due to the rarity of TLS in solid tumors and in the absence of chemotherapeutics. After initiating treatment, beginning on day nine, uric acid and potassium were better controlled. Creatinine kept rising, indicating the need for hemodialysis, which the patient declined. BUN- blood urea nitrogen; LDH- lactate dehydrogenase *Bolded values represent significantly abnormal lab results

Day	Uric Acid	Phosphorus	Potassium	BUN	Creatinine	Calcium	LDH
1	-	-	5.4	52	2	10.4	-
2	-	-	4.3	56	2.4	10	-
3	-	4.8	4.5	59	3	9.9	-
4	-	-	4.7	66	3.6	9.4	-
5	-	-	4.8	72	3.7	9.4	-
6	13.9	5.2	5.2	71	3.8	9.5	641
7	14.2	5.2	5.6	78	4.8	9.8	702
8	-	-	5.3	81	5.4	9.9	-
9	15.8	6	5.8	88	6	9.3	771
10	9.4	6.1	5.1	88	6.1	9.5	772
11	6	6.5	5	88	6.4	9	873
12	4.7	6.8	4.7	95	6.7	9.1	1019
13	5.3	6.5	5.1	99	7	8.6	1255
14	-	-	-	-	-	-	-
REFERENCE RANGE	2.7-7.3 mg/dL (female)	2.8-4.5 mg/dL	3.5-5 mEq/L	6-20 mg/dL	0.6-1.1 mg/dL	8.5-10.5 mg/dL	105-333 U/L

Initially, rasburicase was not immediately started, as the patient had no evidence of any hematologic malignancy, and TLS is rarely known to be caused by solid tumors and is even less likely to occur spontaneously. However, by hospital day 9, her TLS labs continued to progress, so the decision was made to start the patient on lactated Ringer's at 75 cc/hr, rasburicase 6 mg IV PRN, sodium bicarbonate 650 mg po q8, lokelma 10 g daily, and sevelamer 800 mg po q8 to treat the likely STLS. After treatment, the patient’s uric acid levels returned to normal levels, but her creatinine continued to rise, and hemodialysis was recommended. A goals-of-care discussion revealed that comfort and quality of life were most important to the patient. As such, she decided not to proceed with hemodialysis or chemotherapy and sought instead to continue inpatient hospice care with comfort measures only. The patient died 14 days after her initial presentation to the hospital.

## Discussion

This is not the first case report describing STLS in a lung neuroendocrine tumor. A case report and literature review written in 2018 found four such cases of STLS resulting from neuroendocrine tumors of the lung [8]. By 2023, two separate literature reviews identified nine cases of STLS secondary to small cell lung cancer (SCLC), a type of neuroendocrine tumor of the lung, and eight of these patients also had liver metastases [2,3]. Perhaps liver metastasis plays a role in the development of STLS secondary to solid tumors. More research is needed to make such a determination. Of the nine patients described, only three survived, underscoring the increased mortality for these patients [2,3].

The only lab finding inconsistent with the textbook picture of TLS in our case was the patient's calcium being consistently WNL; however, this may be explained by competing calcium derangements; TLS reduces calcium, while hypercalcemia of malignancy due to the bony metastasis raises it [9]. Although, as the patient's condition deteriorated, calcium levels slowly decreased throughout the hospital stay.

While there is no direct evidence that a biopsy can trigger TLS, there is some anecdotal evidence [2,10]. In our case, the patient underwent a biopsy, but it remains unclear if TLS developed before the biopsy or in response to it, since we only have TLS labs in the days following the biopsy. More research is necessary to study the potential risk posed by a biopsy.

With increasing evidence of STLS in neuroendocrine tumors of the lung and other solid tumors, it may be appropriate to periodically monitor these patients' relevant lab values, including uric acid, potassium, calcium, phosphorus, LDH, BUN, and creatinine. Perhaps in patients with high tumor burden, who are at an increased risk for STLS [2], even prophylactic allopurinol or rasburicase (depending on uric acid levels) should be administered? After all, the best treatment for TLS is prevention [7]. Earlier detection may also be aided by modified diagnostic criteria for STLS, which obviously would not include timelines relating to chemotherapeutic administration. The absence of clear diagnostic criteria in STLS may also be responsible for an underestimation of incidence. Further research should be conducted to establish appropriate diagnostic criteria, screening methods, and prophylaxis for STLS.

Upon retrospective review of this case, the utility of empiric treatment was heavily considered for future cases similar to this one. In a patient presenting with evidence of progressive metastatic disease for the first time, empiric treatment of STLS should be considered if in line with the patient’s goals of care. While allopurinol's and rasburicase's common side effect profiles may be relatively mild, they still pose a risk of more serious adverse reactions [10]. These risks may ultimately be acceptable for some patients; however, our patient wished to be made comfortable, recognizing the progressive nature of her disease. What was most important to her was comfort and leaving the hospital. For other patients whose goals include medical management and attempts at curative treatment, empirically treating for STLS if suspected may be appropriate if the benefits outweigh the risks and a collaborative decision is made between the patient and interdisciplinary team.

## Conclusions

This case highlights the importance of maintaining a high index of suspicion for STLS in patients with solid tumors, particularly pulmonary neuroendocrine tumors with extensive metastatic disease. Although TLS is classically associated with hematologic malignancies and chemotherapy, emerging evidence demonstrates that STLS can occur in solid tumors and carries a high risk of morbidity and mortality. Our patient’s course underscores the need for early recognition, prompt initiation of supportive measures, and consideration of prophylactic monitoring in high-risk individuals; furthermore, the potential role of liver metastases in precipitating STLS of solid tumors warrants further investigation. Clinicians should remain vigilant for this rare yet life-threatening complication, as timely diagnosis and intervention may improve outcomes in an otherwise rapidly fatal condition.
